# Cortical actin networks induce spatio-temporal confinement of phospholipids in the plasma membrane – a minimally invasive investigation by STED-FCS

**DOI:** 10.1038/srep11454

**Published:** 2015-06-29

**Authors:** Débora M. Andrade, Mathias P. Clausen, Jan Keller, Veronika Mueller, Congying Wu, James E. Bear, Stefan W. Hell, B. Christoffer Lagerholm, Christian Eggeling

**Affiliations:** 1 Department of Nanobiophotonics, Max Planck Institute for Biophysical Chemistry, Am Fassberg 11, Göttingen 37077, Germany; 2 MRC Human Immunology Unit and Wolfson Imaging Centre Oxford, Weatherall Institute of Molecular Medicine, University of Oxford, Headley Way, Oxford OX3 9DS, UK; 3 Centre for Neural Circuits and Behaviour, University of Oxford, Mansfield Road, Oxford OX1 3SR, UK; 4 MEMPHYS - Center for Biomembrane Physics, University of Southern Denmark, Campusvej 55, Odense M,DK-5230, Denmark; 5 Department of Cell & Developmental Biology, and Lineberger Comprehensive Cancer Center, University of North Carolina, Chapel Hill NC 27599, USA; 6 Howard Hughes Medical Institute, University of North Carolina, Chapel Hill NC 27599, USA

## Abstract

Important discoveries in the last decades have changed our view of the plasma membrane organisation. Specifically, the cortical cytoskeleton has emerged as a key modulator of the lateral diffusion of membrane proteins. Cytoskeleton-dependent compartmentalised lipid diffusion has been proposed, but this concept remains controversial because this phenomenon has thus far only been observed with artefact-prone probes in combination with a single technique: single particle tracking. In this paper, we report the first direct observation of compartmentalised phospholipid diffusion in the plasma membrane of living cells using a minimally invasive, fluorescent dye labelled lipid analogue. These observations were made using optical STED nanoscopy in combination with fluorescence correlation spectroscopy (STED-FCS), a technique which allows the study of membrane dynamics on a sub-millisecond time-scale and with a spatial resolution of down to 40 nm. Specifically, we find that compartmentalised phospholipid diffusion depends on the cortical actin cytoskeleton, and that this constrained diffusion is directly dependent on the F-actin branching nucleator Arp2/3. These findings provide solid evidence that the Arp2/3-dependent cortical actin cytoskeleton plays a pivotal role in the dynamic organisation of the plasma membrane, potentially regulating fundamental cellular processes.

The conceptualisation of the Singer-Nicholson fluid mosaic model for biological membranes^1^ is a milestone in membrane research. Nevertheless, novel methods for probing membrane dynamics have brought a wealth of insight that contradicts this model. Particularly, the assumption that proteins and lipids undergo Brownian diffusion in the plasma membrane has been shown to be largely inaccurate^2^,^3^,^4^,^5^,^6^,^7^,^8^. On the contrary, different methods have shown that the lateral motion of membrane molecules is constrained by different mechanisms. Generally, such constraints have been attributed to different membrane-organising principles; 1) interactions with transient self-assemblies of specific lipids and lipid-anchored proteins, the so-called “lipid rafts”^7^,^9^,^10^, 2) direct or indirect interactions with actin-cytoskeleton associated barriers or anchors (such as cytoskeleton-anchored proteins)^3^,^4^,^6^,^11^,^12^,^13^,^14^,^15^, or 3) membrane curvature^16^,^17^. Concerning the second point, it has been shown by different methods that a variety of membrane proteins are constrained by the actin cytoskeleton^12^,^14^,^15^. In addition, SPT experiments have suggested that even phospholipid diffusion in the plasma membrane is constrained, presumably also by the cortical actin cytoskeleton^2^,^18^. In view of these findings, the “picket-fence” model was proposed^3^. This model hypothesises that direct anchoring of transmembrane proteins (the pickets) to cortical cytoskeletal filaments (the fences) directly beneath the plasma membrane create restrictive barriers, and that these barriers indirectly constrain the diffusion of other membrane proteins and of lipids (Fig. 1a). Specifically, these barriers create compartments within the plasma membrane in which molecules can diffuse freely, while crossing from one compartment to the next is constrained, resulting in compartmentalised or “hop”-diffusion. The premise of compartmentalisation of membrane proteins and lipids is a very attractive proposition because it may be associated with for example localised signalling^3^. In this context, diffusion of integral membrane proteins within compartments was shown to enhance the interaction probability of less abundant proteins, thereby potentially triggering important cellular events^14^,^15^.

The picket-fence model for compartmentalisation of phospholipids in the plasma membrane has encountered several obstacles for its full acceptance^8^,^19^,^20^. Principally, compartmentalised phospholipid diffusion has thus far only been observed by SPT experiments in which gold particles^2^ and quantum dots (QDs)^18^ were employed in order to access the sub-millisecond temporal resolution regime that is required. However, these probes are very artefact-prone due to their prominent size, and due to the difficulty in validating the probe valence for the target molecules (Fig. 1b). Thus, it cannot be ruled out that these probes do neither affect the native target molecule mobility by steric hindrance nor induce target molecule oligomerisation^2^,^20^. In addition, the validity of SPT reports on compartmentalised diffusion was drawn into question by a study showing that the irregularity of plasma membrane topography can induce an artificial observation of compartmentalised diffusion by this technique^21^.

In order to resolve the dilemma regarding actin cytoskeleton-modulated lipid compartmentalised diffusion, we have applied stimulated emission depletion fluorescence correlation spectroscopy (STED-FCS)^5^,^22^,^23^ to probe the diffusion of a phospholipid analogue, labelled with a small and potentially less invasive organic dye, in the plasma membrane of living cells (Fig. 1b). STED-FCS allows for a systematic probing of molecular diffusion for observation spot sizes ranging from a diffraction-limited 240 nm down to below 40 nm, a range that is comparable in size to the postulated actin cytoskeleton-mediated compartments^2^,^3^,^4^. In the current experiments, we have used the phospholipid di-palmitoyl-phosphoethanolamine (DPPE) labelled with the fluorescent dye Atto647N at the head group. The label has been shown to change some of the lipids characteristics, such as changed its preference from more ordered to more disordered membrane environments^23^,^24^,^25^. However, the Atto647N dye modification has been found, by STED-FCS, to have no interfering effect upon the diffusion dynamics and lipid-lipid interactions in living cells^5^,^23^. Previous STED-FCS investigations in live PtK2 and HeLa cells have further shown that diffusion of this DPPE analogue is mainly free and hardly hindered by transient interactions with other membrane molecules such as slow-moving or cytoskeleton-anchored proteins or by membrane curvature^5^,^23^,^24^. For these reasons, we believe that this lipid analogue is a very good candidate for delineating the effect on phospholipid diffusion due to cortical cytoskeleton-dependent membrane partition from that due to other hindrances.

Here, the experimental studies were conducted in two adherent cell types: 1) NRK fibroblasts, and 2) Ink4a/Arf (−/−) mouse embryo fibroblasts (IA32 MEFs)^26^. These cell types were selected because previous work using SPT in combination with either gold particles or QDs as lipid labels has suggested that phospholipid diffusion in these cell types is compartmentalised^2^,^18^. Both of these cell types furthermore contain very distinct actin-rich regions either in-between a distinct stress-fibre network supporting strong adhesion sites to the cover glass surface (NRK), or in the form of prominent lamellipodia followed by large, thin lamella (IA32)^26^. The current studies show for the first time that STED-FCS enables the observation of compartmentalised lipid diffusion, and furthermore that even minimally invasive phospholipid probes are spatially constrained within compartments of the plasma membrane.

## Results

### Resolving Compartmentalised Diffusion

**Figure 2:**
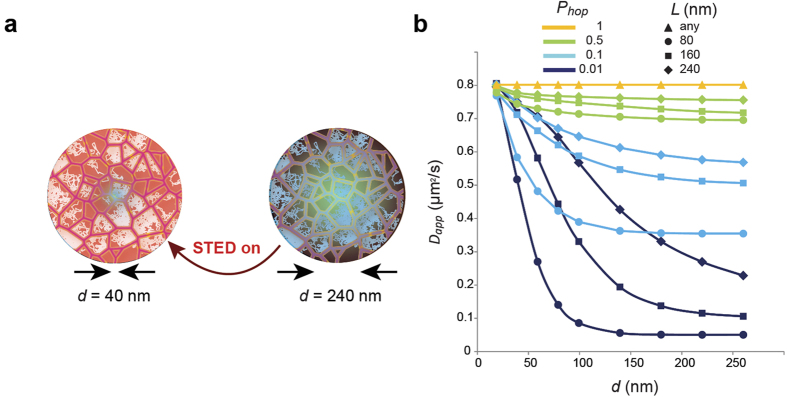
STED-FCS simulation of compartmentalised diffusion. (**a**) In STED-FCS, the apparent diffusion coefficient *D*_*app*_ is determined for different sizes of the observation spot (given by the diameter *d*), as formed by varying the STED laser power (red: STED light, green: effective observation or fluorescence area). (**b**) *In silico* STED-FCS experiments: Simulations show characteristic dependencies of *D*_*app*_ on the diameter *d* of the observation area, assuming a model for compartmentalised diffusion as depicted in Fig. 1a with *D*_free_ = 0.8µm^2^/s. As *d* is increased, *D*_*app*_ decreases. Characteristic compartment size of length *L*, free diffusion coefficient *D*_*free*_, and hopping probability *P*_*hop*_ define the diffusion model. These simulations (using *D*_free_ = 0.8µm^2^/s and *P*_*hop*_ and *L* as given) show that only strong confinement (small *P*_*hop*_) renders clear patterns of compartmentalised diffusion whereas weaker confinement (for example, *P*_*hop*_ = 0.5) closely resembles free diffusion.

To demonstrate the applicability of STED-FCS for detecting compartmentalised diffusion, we first simulated diffusion within a heterogeneous lattice with a characteristic average compartment length, *L, in silico*. Molecules were assumed to diffuse freely within compartments with a diffusion coefficient *D*_*free*_, while transposing compartment boundaries was possible with a certain “hopping probability”, *P*_*hop*_. The simulated trajectories were transformed into intensity traces, which were auto-correlated to generate *in silico* FCS curves for different observation spots. These curves were then fitted using standard STED-FCS analysis, which determines the average apparent diffusion coefficient, *D*_*app*_, of the molecules as a function of the observation spot diameter, *d*^23^ (Fig. 2). The resulting dependence of the apparent diffusion coefficient, *D*_*app*_, on the diameter, *d*, of the observation spot reveals the spatial constraints of molecular diffusion^4^,^23^.

In these simulations, free diffusion (*P*_*hop*_ = 1) is characterized by a *D*_*app*_ that is independent of *d*, whereas compartmentalised diffusion (*P*_*hop*_ < 1) is highlighted by a decrease of *D*_*app*_ with increasing *d*. Measurements at small observation spots primarily probe the free diffusion within the compartments (*D*_*app*_* = D*_*free*_), while measurements at observation spots that are comparable to or larger than the compartments themselves result in a reduced apparent diffusion coefficient, *D*_*app*_* < D*_*free*_, because the compartment barriers slow down the molecular transits through the observation spot. Specifically for *P*_*hop*_ « 1, i.e. strong confinement, compartmentalised diffusion can be clearly distinguished from free diffusion using STED-FCS (Fig. 2).

### Compartmentalised Diffusion of Lipids Observed by STED-FCS in NRK and IA32 Cells

**Figure 3:**
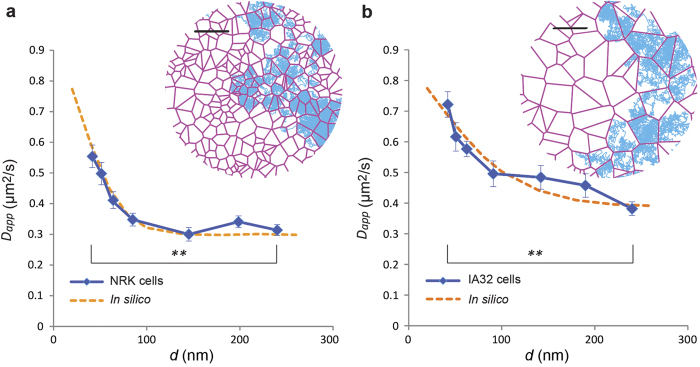
Experimental observation of lipid compartmentalised diffusion by STED-FCS. *D*_*app*_(*d*) dependencies (blue) for DPPE-Atto647N diffusion in NRK (**a**) and IA32 cells (**b**). Clear compartmentalised diffusion patterns are observed. **P < 0.01 (unpaired *t* test). Error bars are s.e.m. In *a*, *n* = 32 cells; in *b*, n = 33 cells. In *a* and *b*, *r* = 10 (*n* stands for the number of cells, from *r* samples). Fitting of the experimental data using Monte-Carlo simulations (orange dotted lines) resulted for NRK cells, *D*_free_ = 0.8 ( ±0.03) μm^2^/s, *P*_hop_ = 0.1 ( ±0.01) and *L* = 80 ( ±8) nm, and for IA32 cells, *D*_free_ = 0.8 ( ±0.02) μm^2^/s, *P*_hop_ = 0.1 ( ±0.01) and *L* = 150 ( ±12) nm. Insets: Representative Voronoi lattices (red) relative to the correspondent compartment sizes as well as simulated diffusion trajectories (blue) correspondent to the fitted parameters. Scale bars: 250 nm.

Experimental STED-FCS measurements (Fig. S1) were conducted in NRK fibroblasts and IA32 MEFs. In both cell types, we principally measured diffusion at thin peripheral areas of the cells (within 2–5 μm from the cell edge), resulting in combined measurements of both apical and basal membranes (Fig. S2a). These measurements resulted in a clear pattern of compartmentalised diffusion as indicated by a significant decrease of *D*_*app*_(*d*) at large observation diameters (Fig. 3). The experimentally observed dependencies of *D*_*app*_ on *d* were fit using Monte Carlo simulations to a model of free diffusion constrained by a heterogeneous lattice. The fitting parameters that best described the data were for NRK cells, *D*_free_  = 0.8 (±0.03)μm^2^/s, *P*_hop_  = 0.1 (±0.01) and *L*  = 80 (±8)nm, and for IA32 cells, *D*_free_  = 0.8 (±0.02)μm^2^/s, *P*_hop_  = 0.1 (±0.01) and *L*  = 150 (±12)nm.

### Pharmacological Modulation of the Arp2/3 Complex Specifically Impacts Lipid Compartmentalised Diffusion

**Figure 4:**
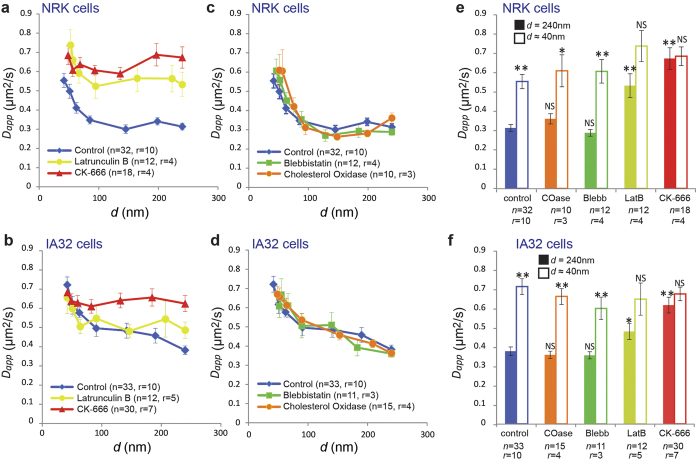
Investigation of molecular mechanisms underlying compartmentalised lipid diffusion via STED-FCS: Pharmacological treatments. *D*_*app*_(*d*) dependencies: (**a,b**) Cytoskeleton depletion in NRK (**a**) and IA32 (**b**) cells, respectively: treatment with Latrunculin B and CK-666 as labelled, (**c,d**) Cholesterol depletion and myosin II inhibition in NRK (**c**) and IA32 (**d**) cells, respectively: treatment with Cholesterol Oxidase and Blebbistatin as labelled. (**e,f**) Summary of NRK and IA32 data, respectively, showing values of *D*_*app*_ for confocal (*d* = 240 nm, filled columns) and STED recordings (*d* ~ 40 nm, open columns) – the increase in *D*_*app*_ from 240 to 40 nm indicates the extent of compartmentalised diffusion. Error bars are s.e.m. Symbols on top of the columns represent results of the statistical test (**P < 0.01, *P < 0.05, NS not significant; two-tailed unpaired *t* test): for *d *~ 40 nm comparison with the value representing *d* = 240 nm in the same experiment, and for *d* = 240 nm comparison with the respective value for *d* = 240 nm in the control (untreated) experiment. *n* stands for the number of cells, from *r* samples.

In order to assess the underlying molecular mechanisms for the observed pattern of compartmentalised diffusion, we systematically performed STED-FCS experiments on NRK and IA32 cells where the 1) actin cytoskeleton was modulated, 2) membrane cholesterol was depleted, or 3) myosin II activity was inhibited (Fig. 4).

Actin cytoskeleton modulation was achieved by treating cells with either latrunculin B (LatB) or CK-666^27^,^28^. LatB treatment globally inhibits the polymerization of all F-actin networks, whereas CK-666 specifically inhibits the actin nucleator Arp2/3^29^ and consequently reduces the cytoskeleton branching. The diffusion of the DPPE analogue following LatB treatment was faster at *d* = 240 nm in both cell types, markedly in NRK cells (Fig. 4a,b), but weak compartmentalised diffusion was still observed as was determined by comparing the measurements of *D*_*app*_ from STED-FCS measurements at *d* = 240 nm and *d *≈ 40 nm with an unpaired Student t-test (NRK *P* = 0.06; IA32 *P* = 0.06) (Fig. 4a,b and Table S1). The diffusion of DPPE in cells treated with CK-666 was remarkably faster at *d* = 240 nm in both cell types, and compartmentalised diffusion was no longer observed (NRK *P* = 0.87; IA32 *P* = 0.42) (Fig. 4a,b and Table S1).

Conversely, blebbistatin inhibition of myosin II had no effect on DPPE diffusion in neither NRK nor IA32 cells (Fig. 4c,d and Table S1). This result suggests that the observed compartmentalised diffusion was independent of myosin-based contractility. Similarly, cholesterol depletion using cholesterol oxidase (COase) also had no effect on DPPE diffusion (Fig. 4c,d and Table S1), indicating that compartmentalisation of DPPE was independent of cholesterol-mediated interactions. This strongly suggests that the observed compartmentalised diffusion of DPPE is dependent on the cortical actin cytoskeleton and is specifically modulated by Arp2/3-dependent cortical actin networks.

**Figure 5:**
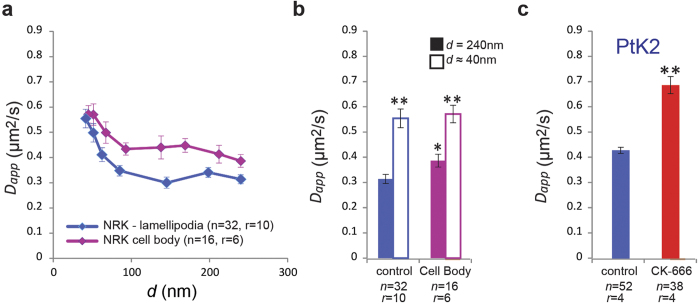
STED-FCS of lipid diffusion in different parts of the cell and in PtK2 cells. (**a**) *D*_*app*_(*d*) dependencies in NRK cells: diffusion near the cell edge (lamellipodia) and under the cell body (as labelled). (**b**) Values of *D*_*app*_ for confocal (*d* = 240 nm, filled columns) and STED recordings (*d* ~ 40 nm, open columns) for diffusion probed near the cell edge (control, blue) and under the cell body (purple) – the increase in *D*_*app*_ from 240 to 40 nm indicates the extent of compartmentalised diffusion. Compartmentalised diffusion is more pronounced near the cell edge. (**c**) Results from measurements on PtK2 cells: Experimental diffusion coefficient of the fluorescent DPPE analogue in the plasma membrane of untreated and CK-666-treated PtK2 cells (*d* = 240 nm). We have previously shown^5^,^23^ that diffusion of this DPPE analogue in PtK2 cells is apparently free, therefore the measurements corresponding to *d* = 240 nm are expected to effectively represent the range (*d* = 40 nm to *d* = 240 nm). Error bars are s.e.m. Symbols on top of the columns represent results of the statistical test (**P < 0.01, *P < 0.05, NS not significant; two-tailed unpaired *t* test): for *d* ~ 40 nm comparison with the value representing *d* = 240 nm in the same experiment, and for *d* = 240 nm comparison with the respective value for *d* = 240 nm in the control experiment. Here, *n* stands for the number of cells, from *r* samples.

To assess the generality of the observed actin cytoskeleton modulation of lipid diffusion, we also performed STED-FCS measurements closer to the cell body of NRK cells. Unlike IA32 cells, NRK cells feature relatively large cell bodies, where STED-FCS specifically probes the basal plasma membrane (Fig. S2b). These measurements resulted in the observation of faster and slightly less compartmentalised diffusion, as compared to the measurements near the cell edge, which combine effects of diffusion in the basal and apical plasma membranes (Fig. 5a,b and Table S1). This can be explained either by a less branched F-actin network beneath the cell body, as compared to the cell edge, or by a less branched F-actin network in the basal plasma membrane, as compared to the apical membrane^30^.

While compartmentalised diffusion of DPPE was clearly observed in IA32 and NRK cells, our previous STED-FCS measurements in PtK2 cells using the same fluorescent DPPE analogue was indicative of free diffusion^5^,^23^. Following CK-666 treatment, we now however also confirmed for PtK2 cells that diffusion of DPPE was significantly faster than in untreated cells, revealing that lipid diffusion in this cell type is also compartmentalised by Arp2/3-dependent cortical actin networks (Fig. 5c). This then suggests that: either the compartment size, *L*, in unperturbed PtK2 is smaller than the accessible spatial sampling range of STED-FCS, or that the compartment strength, *P*_*hop*_, is weaker than that seen here in the case of NRK cells and IA32 MEFs. Application of our simulation model to the case of PtK2 cells, we now find that a combination of these factors can explain the previous observation of apparent free diffusion of DPPE in PtK2 cells, as our simulation model shows that compartmentalised diffusion with *D*_*free*_ = 0.7 μm^2^/s is perceived as free diffusion (for *d* > 40 nm) with *D*_*app*_ = 0.4 μm^2^/s if, for example, *L* = 25 nm and *P*_*hop*_ = 0.25 (Fig. S3).

### Validation of the Role of the Arp2/3 Complex in Lipid Compartmentalisation in Arp2/3 Depleted MEFs

**Figure 6:**
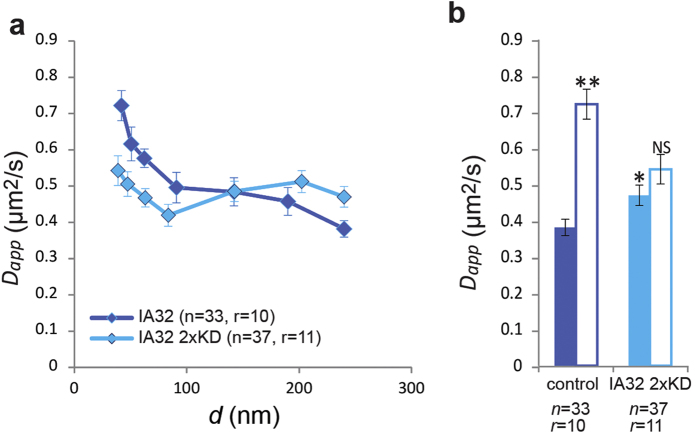
Comparing compartmentalisation in Arp2/3 knock-down cells. (**a**) *D*_*app*_(*d*) dependencies in IA32 MEFs and IA32 2xKD MEFs (Arp 2/3 knock-down), respectively. (**b**) Values of *D*_*app*_ for confocal (*d* = 240 nm, filled columns) and STED recordings (*d* ~ 40 nm, open columns) in IA32 MEFs (control, dark blue) and IA32 2xKD MEFs (IA32 2xKD, light blue) – the increase in *D*_*app*_ from 240 to 40 nm indicates the extent of compartmentalised diffusion. Error bars are s.e.m. Symbols on top of the columns represent results of the statistical test (**P < 0.01, *P < 0.05, NS not significant; two-tailed unpaired *t* test): for *d* ~ 40 nm comparison with the value representing *d* = 240 nm in the same experiment, and for *d* = 240 nm comparison with the respective value for *d* = 240 nm in the control experiment. *n* stands for the number of cells, from *r* samples.

In order to validate the effects of Arp2/3 depletion, we also performed experiments in IA32 MEFs that had been additionally depleted of p34Arc and Arp2, two essential subunits of the Arp2/3 complex (IA32 2xKD)^26^. These cells are characterized by the absence of lamellipodia and, as demonstrated by electon microscopy, a much sparser actin network in the lamella that culminates in frequent filopodia at the cell edge^26^. STED-FCS measurements in these cells were as before acquired in thinner regions at the periphery of the cells. The resulting dependence of *D*_*app*_(*d*) in these cells is compatible with free diffusion (*P* = 0.15) (Fig. 6 and Table S1). This result corroborates the pivotal role of Arp2/3 in restricting lipid diffusion in the plasma membrane. For small observation areas, we also observed that DPPE diffusion was slower in IA32 2xKD MEFs than in either WT or CK-666 treated IA32 MEFs. We speculate that the reason for this could be related to increased membrane curvature as a consequence of less sub-membranous cortical actin mechanical support – a speculation supported by the phenotypic observation that the IA32 2xKD MEFs have a defective cell volume response to osmotic stress challenges^31^. Unfortunately, no suitable methods to probe the relation between hindered diffusion and membrane curvature in live cells exist so far.

## Discussion

In this work, STED-FCS facilitated a minimally invasive observation of fluorescent phospholipid analogue diffusion in the plasma membrane of living cells. Our data showed a clear decrease of the apparent diffusion coefficient with increasing observation spot sizes, suggesting that phospholipids undergo compartmentalised diffusion in the plasma membrane. The confinement strength was observed to be similar in both investigated cell types such that on average one out of ten collisions with the confinement barrier resulted in the escape of the lipid probe from confinement regions of size *L*, i.e. *P*_*hop*_ = 0.1. *L* was found to be about two-fold smaller in NRK cells (*L* = 80 nm) than in IA32 MEFs (*L* = 150 nm). The unhindered free diffusion coefficient within the confinement regions was in both cases the same of about *D* = 0.8 μm^2^/s. Applying the Saffman-Delbruck model^32^ to these observations, this then suggests that the viscosity of the plasma membrane within the confinement regions was identical in both cell types.

Using a combination of pharmacological treatments, we showed that this temporal confinement was dependent on the actin cytoskeleton, but not on the plasma membrane cholesterol content. Recent studies have shown that the actin cytoskeleton is not a single structure but is composed of distinctly different sub-structures^28^. Most relevant to this work is the cortical actin cytoskeleton which is the actin network immediately below the plasma membrane, and which directly couples to the membrane via a variety of actin binding proteins and cytoplasmic domains of certain membrane proteins^33^. Unfortunately, definitive fluorescence imaging of the cortical cytoskeleton was currently not possible in cells under physiological conditions due to limited axial resolution. However, our results revealed with unprecedented detail that the cortical actin effectively constrained phospholipid diffusion thus causing compartmentalised diffusion in the plasma membrane.

Unlike other cytoskeletal structures, the composition of the cell cortex is poorly understood^34^. Enhancing the knowledge about this important structure, this study definitively pinpoints that the Arp2/3 complex as a component of the cortical actin networks thus also confirming previous studies^31^,^35^,^36^. Arp2/3 is already an acknowledged critical component in the generation of dense, highly branched F-actin networks at the leading edge of lamellipodia and/or at adhesion sites^26^,^37^, and orchestrates numerous tasks performed by the actin cytoskeleton^29^. Recent studies using the Arp2/3 inhibitor CK-666 and Arp2/3-depleted mammalian cells have also revealed new roles of Arp2/3-branched actin network in a variety of cellular processes including matrix sensing, cytoplasmic streaming, spindle positioning and cell-cell junction regulation^26^,^29^,^31^,^36^,^38^,^39^,^40^. This study adds further functions to the Arp2/3 complex by providing conclusive experimental evidence that the Arp2/3 complex also regulates cortical actin meshwork branching and that this directly affects phospholipid diffusion within the plasma membrane.

The pattern of compartmentalised diffusion observed in our studies is in partial agreement with the original gold probe-based SPT studies by Kusumi and co-workers that first suggested that the diffusion of lipids in the plasma membrane of NRK cells is restricted by the actin cytoskeleton^2^. In particular, the molecular origin of the compartmentalisation is in both instances directly dependent on the actin cytoskeleton but is independent of cholesterol content. However, our results in NRK cells show a much slower apparent diffusion coefficient within compartments (0.8 μm^2^/s compared to 5.4 μm^2^/s) and about 2-fold faster long-term diffusion coefficients (0.31 μm^2^/s compared to 0.17 μm^2^/s). This indicates that the plasma membrane compartmentalisation in our STED-FCS studies in NRK cells with a smaller and thus potentially less invasive probe (Atto647N label) is much weaker than it was suggested in the previous SPT studies with a much larger and thus potentially more invasive gold probe. Additionally, our results for the long-term diffusion coefficient are in much closer agreement with previous SPT measurements using a comparably minimally invasive dye labelled Cy3-DOPE lipid^2^ but for which compartmentalised diffusion could not be detected due to limited temporal sampling.

The observed discrepancy between our STED-FCS study and previous SPT studies could be explained by the differences in the lipid probes. For example, gold probes and also related QD probes are linked to lipids via biomolecules that can attach several lipids at the same time. Oligomerised structures are likely to be significantly more sensitive to diffusion barriers, which explains the small long-term diffusion coefficient observed with SPT in combination with gold probes and QDs^18^, as compared to our studies with Atto647N-DPPE or those with Cy3-DOPE^2^. Conversely, the very fast short-term diffusion coefficient reported by Kusumi and co-workers is similar to that measured in artificial lipid membranes^3^. However, it is unlikely that lipids should diffuse at the same coefficient in free standing membranes (lipid vesicles) as within sub-compartments of the plasma membrane, considering the potential steric effects caused by protein crowding on lipid diffusion in the plasma membrane as well as the potential friction effects induced by the glycocalyx on the apical plasma membrane of cells. The presented data in NRK cells further cannot be satisfactorily described by the current simulation model if *D*_*free*_ is fixed to 5.4 μm^2^/s. STED-FCS measurements at even smaller observation spots (*d* < 40 nm) would help to define *D*_*free*_ more precisely (Figure S4).

We also observed a discrepancy in the mean compartment size in NRK cells whereby our results were 3-fold smaller (*L* = 80 nm) as compared to previous measurements by SPT (*L* = 230 nm). This difference could be partly explained by the fact that the spatial precision in the SPT measurements is limited by the localisation error such that the size of the compartments will be artificially larger as has been proposed previously^41^. This effect is also likely significant because the typical reported localisation errors of 10–20 nm are in many cases of similar length scales to the measured displacements of single molecules at the short time scales where the confined diffusion is observed. In contrast, the spatial precision in the STED-FCS measurements stems from the precision by which the observation areas can be determined. This precision is always much smaller than the measured observation areas. Thus, we are inclined to believe that the accuracy of our measurements of the size of the confining compartments is better due to much better spatial precision. However, this discrepancy has to be fully validated, preferably by a comparative study of both methods and using identical cell types and lipid analogues.

It has also been suggested that membrane curvature can explain the previously reported hop-diffusion^21^, although this has never been demonstrated to affect STED-FCS experiments. However, to be detectable by STED-FCS the confinement has to be strong, and thus the membrane curvature would have to be unnaturally prominent. The reported STED-FCS measurements are also the average of multiple measurements in multiple cells where each measurement is obtained by random, unbiased positioning of the observation spot on the plasma membrane such that hypothetical extreme curvature effects are likely averaged out. Nevertheless, we cannot completely rule out possible curvature effects, including indirect effects that might originate from changes in curvature in response to our pharmacological treatments of the actin cytoskeleton. Unfortunately, such effects cannot currently be measured. Thus, cortical actin-assisted compartmentalisation provides the most accurate model to describe the general constraints that limit lipid diffusion in the absence of specific molecular interactions.

In summary, we have shown in this work that the cortical actin cytoskeleton directly influenced phospholipid diffusion in the plasma membrane of cells. Furthermore, we have for the first time shown that the Arp2/3 complex is directly involved in this process. Following our observations in three unrelated cell types (NRK, IA32 MEFs, and PtK2), we are compelled to infer that plasma membrane compartmentalisation by the actin cytoskeleton is a fundamental cellular process, generic to most cells. The determination of the precise structural and molecular mechanisms by which the cytoskeleton performs lipid compartmentalisation (for example, by steric effects caused by trans-membrane proteins anchored to cortical actin filaments^2^, possibly accompanied by changes in membrane order^42^), as well as the mechanisms by which it may be implied in fundamental processes such as cell signalling, are exciting open questions that emerge from the still evolving picture of the plasma membrane organisation. Further, we conclude that models for membrane organisation that do not acknowledge the symbiosis between the plasma membrane and the cytoskeleton are oversimplified^12^.

## Methods

### STED-FCS nanoscopy

The STED nanoscope was based on a home-built confocal microscope setup equipped with a 640 nm laser (≈100 ps pulse width, LDH-D-C-640, PicoQuant) for excitation with a repetition rate of 45 MHz of the fluorescent label. The STED beam was provided by a Titanium:Sapphire laser system (Chameleon, Coherent Inc.) operating at 780 nm with a repetition rate of 90 MHz. The time interval between the pulses of both lasers was adjusted using a home-built electronic delay unit, where the STED pulses served as the trigger master. The STED laser pulses were stretched from 200 fs to a pulse length of approximately 180 ps using four 30 cm optical SF6 glass rods and a 125 m long polarisation maintaining single-mode fibre (OZ Optics). Fluorescence excitation and collection was realized using an oil immersion objective (APON 60x, NA = 1.49, Olympus). The laser beams were spatially overlaid and the fluorescence light filtered by appropriate (dichroic) filters (AHF Analysentechnik, Tübingen, Germany). The doughnut-shaped focal spot of the STED beam featuring a central intensity zero was produced by introducing a phase-modifying plate (RPC Photonics) into the beam path, imprinting on the wave front a helical phase ramp exp(iφ) with 0 ≤ φ ≤ 2π. A λ/4-plate ensured circular polarisation of the STED and excitation beams. The fluorescence was coupled into a multi-mode fibre splitter (Fiber Optic Network Technology) with an aperture size corresponding to 1.4x the magnified excitation spot. The 50:50 split fluorescence signal was then detected by two single-photon counting modules (avalanche photo diode SPCM-AQR-13-FC, Perkin Elmer Optoelectronics) and the recorded fluorescence counts were further processed by a hardware correlator card (Flex02-01D, Correlator.com). The focal intensity distribution of the excitation and STED light were measured by scanning a scattering gold bead of 80 nm in diameter (gold colloid, En.GC80, BBinternational) using a non-confocal detector (MP 963 Photon Counting Module, Perkin Elmer). The applied laser powers *P* were measured directly at the sample plane. Together with the full-width-at-half-maximum FWHM of the focal laser intensity distribution, they allow for the calculation of the time-averaged intensity *I* = *P*/[π(FWHM/2)^2^] of the diffraction-limited excitation light (usually ~14 kW/cm^2^ stemming from *P* = 7μW) and a time-averaged maximum intensity *I* = 1/2 *P*/[π(FWHM/2)^2^] at the doughnut-crest of the STED laser (~105 MW/cm^2^ stemming from *P* = 98 mW for the highest STED power). Calibration of the diameter *d*(*P*_*STED*_) of the effective fluorescence observation spots formed by a certain STED power *P*_*STED*_ was performed by STED-FCS measurements of fluorescent lipid analogues in supported lipid bilayers (SLBs), for that such lipid bilayers provide a two-dimensional free diffusing system of molecules, labelled with the same fluorophore used in our cellular experiments. The confocal FWHM being determined by fluorescent beads (*d*(*P*_*STED*_ = 0)≈240 nm), the other effective diameters *d*(*P*_*STED*_≠0) can be calculated using the relation:


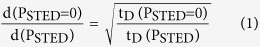


where *t*_*D*_ stands for the average transient times through the observation spot as determined by FCS for each given *P*_*STED*_. The relation above stems from the fact that for absolute two-dimensional free diffusion, the transit time scales proportionally with the size of the observation area.

### STED-FCS analysis

The general theoretical background for STED-FCS analysis has been described in detail^5^,^23^. Correlation data were recorded with lipid concentrations resulting in a temporal average of particle number *N *≈ 0.5–2 fluorescent particles in the observation spot for the highest STED power and *N *≈ 10–30 fluorescent particles for confocal recording. We fitted all correlation data with a model for two-dimensional diffusion, resulting in different values of the average transit time *t*_*D*_ through the observation spot. Each individual measurement resulted in a transit time *t*_*D*_. The correspondent apparent diffusion coefficient *D*_*app*_ = *d*^2^/(8 *t*_*D*_ ln(2)) was calculated based on the knowledge of the diameter *d* of the observation spot, provided by the calibration of the system.

For comparison of the mean *D*_*app*_ between data representing FWHM = 40 nm and FWHM = 240 nm in the same experiment, as well as for comparison of the mean *D*_*app*_ between data representing FWHM = 240 nm and control experiment (FWHM = 240 nm data), we used unpaired student’s *t*-test.

### Supported Lipid Bilayers (SLBs)

Supported lipid bilayers (SLBs) were used to calibrate the STED-FCS setup. The SLBs were prepared based on the following procedure: The lipid DOPC (1,2-dioleoyl-sn-glycero-3-phosphocholine, Avanti) and the fluorescent lipid analogue (DPPE-Atto647N, Atto-Tec) were mixed in organic solvents (Chloroform/MeOH 1:1) at a lipid concentration of ~1 mg/ml. The ratio of labelled lipids per non-labelled ones was approximately 1:10,000. 50 μl of such solution were dropped onto a piranha-cleaned (Femto-RF, Diener Electronic) standard microscope cover glass (diameter 22 mm, no. 1.5 thickness) and spin-coated at 60 Hz for about one minute. The cover glass was then placed in a microscopy chamber and subsequently the dry thin lipid film was rehydrated with 500 μl buffer solution (150 mM NaCl, 10 mM HEPES). Such bilayers were stable for several hours.

### STED-FCS cellular measurements

IA32, NRK, IA32 2xKD and PtK2 cells were seeded on standard glass coverslips (diameter 18 mm, no. 1.5 thickness) to a confluence of about 60% and grown at 37 °C in a water-saturated atmosphere of 5% CO_2_ in air. Incorporation of the fluorescent lipid analogues (DPPE-Atto647N) into the (presumably outer leaflet of the) plasma membrane of the cells was accomplished via BSA coupling, as in detail outlined before [Refs. 5, 23].

We assessed the dynamics of DPPE-Atto647N by placing the focused co-centred excitation and STED beams on random positions at the plasma membrane. On the lamellipodium or very close to the edge of the cells, we probed lipid diffusion concurrently in both the apical and basal membranes. That happens because the effective observation volume is decreased only laterally by the STED beam, remaining approximately 700 nm long in the axial direction (for excitation wavelength of 640 nm), thus encompassing both membranes when they are close enough (Fig. S2). Especially, in the cell body of NRK cells, the measurements were taken at the basal membrane, since the bulky cell body of these cells features a separation between apical and basal membranes (~2 to 7 μm) which ensures STED-FCS experiments to probe only the basal membrane (Fig. S2).

Measurements were taken at room temperature and completed before any significant morphological changes in the cell could occur. The duration of all measurements were 10 s; a correlation time longer than two orders of magnitude times the typical transient time of the labelled lipids through the confocal observation area. For each FWHM (or STED power) the average and standard deviation of the apparent diffusion coefficient was calculated in the following way. First, an average value was determined from 5-8 measurement on different positions of a single cell. The averaged values obtained from *n* individual cells (from r > 4 different sample preparations) were then averaged so that the correspondent error bars reflect mostly the variance among cells. We performed experiments in cells with passage number less than 20 and in samples where cells presented normal morphology. Our cells are once per year tested for mycoplasma.

As detailed previously^5^,^23^, we ensured a non-detectable (at least by STED-FCS) influence of the dye label on the diffusion dynamics, and that the observation times were given by the transit times through the observation spot and not shortened due to photobleaching (by measuring at low enough excitation intensities), and that biasing effects by the excitation or STED light due to photobleaching, heating or other (non-linear) effects and diffusion of non-integrated lipids (or dye tags) could be excluded.

### Drug treatments

#### Cholesterol Oxidase

The cells were treated with 1 U/ml Streptomyces spec. COase (Sigma-Aldrich) in HEPES buffered DMEM (HDMEM) (and washed afterwards in HDMEM) for 30 min under culture conditions. Treatment was performed before the insertion of the fluorescent lipid analogues into the plasma membrane.

#### Blebbistatin

Treatment with 15 μM Blebbistatin (EMD Millipore) in HDMEM (and washed afterwards in HDMEM) for 2 h under culture conditions.

#### Latrunculin B

Treatment with 1 μM (100 nM) Latrunculin B (Sigma-Aldrich) in HDMEM (and washed afterwards in HDMEM) for 15 min under culture conditions was performed for IA32 cells (NRK cells). NRK cells were found to be more sensitive to this drug treatment than IA32 cells, in a way that for these cells reduction of Latrunculin B concentration to 100 nM produced similar results to 1 μM. Treatment was performed before the insertion of the fluorescent lipid analogues into the plasma membrane.

#### CK-666

Treatment with 100 μM CK-666 (EMD Millipore) in HDMEM (and washed afterwards in HDMEM) for 4 h under culture conditions. Treatment was performed before the insertion of the fluorescent lipid analogues into the plasma membrane. In addition, insertion of fluorescent lipid analogues and STED-FCS measurements were carried out in 100 μM CK-666 in HDMEM.

### Monte Carlo simulations for STED-FCS measurements

Monte Carlo simulations were performed using custom written routines in Matlab. In these simulations, we generated fluorescence time traces of 2-dimensional diffusion of fluorescent molecules through an observation spot with a Gaussian-shaped fluorescence detection profile. The area explored by diffusion comprised randomly sized compartments. Within a compartment the molecules were assumed to diffuse freely while crossing from one compartment to another was only possible with a given “hopping probability” *P*_*hop*_. This was implemented in the following way: If free diffusion with diffusion constant *D*_*free*_ would have led the lipid to cross the compartment boundary, the effective transposition would have taken place in only a fraction of such cases (being defined by *P*_*hop*_) while in all other cases the molecule would remain diffusing in the original compartment. The simulation area was a circle of 3 μm diameter and the compartmentalisation of this area was implemented as a Voronoi mesh on a uniform random distribution of seed points. We defined the square root of the average compartment area as the average compartment size or length *L*. The average compartment size, the hopping probability and the free diffusion coefficient completely described our simulation model. In most cases a simulation placed 100 independent molecules in the simulation area and took a time span of 200 s with 20 μs time steps.

The simulated fluorescence time traces were auto-correlated and the correlation curves were fitted to the aforementioned two-dimensional diffusion model. The transient time *t*_*D*_ was converted to an apparent diffusion coefficient using *D*_*app*_ = *d*^2^/(8 *t*_*D*_ ln(2)) with diameter *d* of the observation spot given as the FWHM.

Fitting of the measured *D*(*d*) dependence was achieved by performing repeated simulations on iteratively finer spaced parameter grids based on the minimisation of the squared distance of the mean apparent diffusion coefficients to the measured values. After reaching an approximate accuracy of 1 × 10^−10^ cm^2^/s in *D*_*free*_, 0.01 in *P*_*hop*_ and 5 nm in *L*, the program stopped running the iterations and displayed the most pertinent set of fitted parameters achieved.

Confidence intervals on the best model parameters were calculated by applying the Bootstrap method^43^ assuming that the errors of the measured apparent diffusion coefficients (error bars in Fig. 2) are independently and normally distributed. Hereby, synthetic data values were constructed randomly from the measurement errors and obtained for each best parameter value. From this set of parameter values a symmetric interval containing 70% of these possible values could be extracted. It is displayed as error values in the main text. Additionally a negative correlation between *P*hop** and *L* was observed with the axis of largest error being the ratio of both.

## Additional Information

**How to cite this article**: Andrade, D. M. *et al.* Cortical actin networks induce spatio-temporal confinement of phospholipids in the plasma membrane – a minimally invasive investigation by STED-FCS. *Sci. Rep.* **5**, 11454; doi: 10.1038/srep11454 (2015).

## Supplementary Material

Supplementary Information

## Figures and Tables

**Figure 1:**
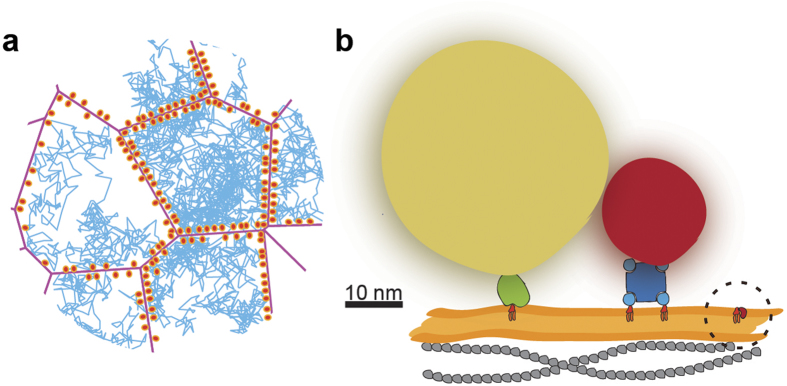
Detecting compartmentalised diffusion with a small lipid probe. (**a**) Schematic showing branched actin networks (magenta) and associated membrane achors (orange), which partially confine two-dimensional diffusion of molecules. As exemplarily shown by single-molecule diffusion tracks (blue), molecules are assumed to diffuse freely within compartments, and in the event of hitting the compartment boundaries, transposition to the adjacent compartment occurs with a certain hopping probability *P*_*hop*_. (**b**) Schematic of lipid probes used in SPT and in STED-FCS, put in perspective (from left to right): gold particle (yellow, ~40 nm in diameter) linked to a lipid (orange: chains, light red: head group) by Fab antibody fragment (green), and QD (red, ~20 nm in diameter) linked to two lipids via streptavidin (blue), as often used in SPT; and a fluorescent lipid analogue (dark red: organic dye, ~1 nm in diameter), as used in STED-FCS. Possible oligomerisation induced by SPT probes is illustrated for the QD. The membrane bilayer is shown in orange and the actin cytoskeleton in grey.

## References

[b1] Singer S. & Nicolson G. L. The fluid mosaic model of the structure of cell membranes. Science 175, 720–731 (1972). 4333397 10.1126/science.175.4023.720

[b2] Fujiwara T., Ritchie K., Murakoshi H., Jacobson K. & Kusumi A. Phospholipids undergo hop diffusion in compartmentalized cell membrane. J Cell Biol 157, 1071–1081 (2002). 12058021 10.1083/jcb.200202050 PMC2174039

[b3] Kusumi A. *et al.* Paradigm shift of the plasma membrane concept from the two-dimensional continuum fluid to the partitioned fluid: High-speed single-molecule tracking of membrane molecules. Annu Rev Biophys 34, 351–378 (2005). 10.1146/annurev.biophys.34.040204.144637 15869394

[b4] Wawrezinieck L., Rigneault H., Marguet D. & Lenne P. F. Fluorescence correlation spectroscopy diffusion laws to probe the submicron cell membrane organization. Biophys J 89, 4029–4042 (2005). 16199500 10.1529/biophysj.105.067959 PMC1366968

[b5] Eggeling C. *et al.* Direct observation of the nanoscale dynamics of membrane lipids in a living cell. Nature 457, 1159–1162 (2009). 19098897 10.1038/nature07596

[b6] Jacobson K., Sheets E. D. & Simson R. Revisiting the fluid mosaic model of membranes. Science 268, 1441–1442 (1995). 7770769 10.1126/science.7770769

[b7] Lingwood D. & Simons K. Lipid Rafts As a Membrane-Organizing Principle. Science 327, 46–50 (2010). 20044567 10.1126/science.1174621

[b8] Wieser S., Moertelmaier M., Fuertbauer E., Stockinger H. & Schutz G. (Un)Confined Diffusion of CD59 in the Plasma Membrane Determined by High-Resolution Single Molecule Microscopy. Biophys.J. 92, 3719–3728 (2007). 17325009 10.1529/biophysj.106.095398 PMC1853144

[b9] Dietrich C., Yang B., Fujiwara T., Kusumi A. & Jacobson K. Relationship of lipid rafts to transient confinement zones detected by single particle tracking. Biophys J 82, 274–284 10.1016/S0006-3495(02)75393-9 (2002). 11751315 PMC1302468

[b10] Jacobson K., Mouritsen O. G. & Anderson R. G. Lipid rafts: at a crossroad between cell biology and physics. Nature Cell Biology 9, 7–14 10.1038/ncb0107-7 (2007). 17199125

[b11] Edidin M., Kuo S. C. & Sheetz M. P. Lateral movements of membrane glycoproteins restricted by dynamic cytoplasmic barriers. Science 254, 1379–1382 (1991). 1835798 10.1126/science.1835798

[b12] Gowrishankar K. *et al.* Active remodeling of cortical actin regulates spatiotemporal organization of cell surface molecules. Cell 149, 1353–1367 (2012). 22682254 10.1016/j.cell.2012.05.008

[b13] Kwik J. *et al.* Membrane cholesterol, lateral mobility, and the phosphatidylinositol 4,5-bisphosphate-dependent organization of cell actin. PNAS 100, 13964–13969 (2003). 14612561 10.1073/pnas.2336102100 PMC283529

[b14] Andrews N. L. *et al.* Actin restricts FcεRI diffusion and facilitates antigen-induced receptor immobilization. Nature Cell Biology 10, 955–963 (2008). 10.1038/ncb1755 PMC3022440 18641640

[b15] Jaqaman K. *et al.* Cytoskeletal Control of CD36 Diffusion Promotes Its Receptor and Signaling Function. Cell 146, 593–606 (2011). 21854984 10.1016/j.cell.2011.06.049 PMC3160624

[b16] Hatzakis N. S. *et al.* How curved membranes recruit amphipathic helices and protein anchoring motifs. Nature Chemical Biology 5, 835–841 10.1038/nchembio.213 (2009). 19749743

[b17] Roux A. *et al.* Role of curvature and phase transition in lipid sorting and fission of membrane tubules. The EMBO Journal 24, 1537–1545 10.1038/sj.emboj.7600631 (2005). 15791208 PMC1142567

[b18] Clausen M. P. & Lagerholm B. C. Visualization of plasma membrane compartmentalization by high-speed quantum dot tracking. Nano Letters 13, 2332–2337 (2013). 23647479 10.1021/nl303151f

[b19] Abbott A. Cell biology: hopping fences. Nature 433, 680–683 (2005). 15716923 10.1038/433680a

[b20] Clausen M. & Lagerholm B. C. The Probe Rules in Single Particle Tracking. Current Protein and Peptide Science 12, 699–713 (2011). 22044141 10.2174/138920311798841672

[b21] Adler J., Andrew I. S., Novak P., Korchev Y. E. & Parmryd I. Plasma membrane topography and interpretation of single-particle tracks. Nature Methods 7, 170–171 (2010). 20195248 10.1038/nmeth0310-170

[b22] Kastrup L., Blom H., Eggeling C. & Hell S. W. Fluorescence Fluctuation Spectroscopy in Subdiffraction Focal Volumes. Phys Rev Lett 94, 178104 10.1103/PhysRevLett.94.178104 (2005). 15904340

[b23] Mueller V. *et al.* STED nanoscopy reveals molecular details of cholesterol- and cytoskeleton-modulated lipid interactions in living cells. Biophys J 101, 1651–1660 (2011). 21961591 10.1016/j.bpj.2011.09.006 PMC3183802

[b24] Honigmann A. *et al.* Scanning STED-FCS reveals spatiotemporal heterogeneity of lipid interaction in the plasma membrane of living cells. Nature Communications 5, 5412 10.1038/ncomms6412 (2014). 25410140

[b25] Sezgin E. *et al.* Partitioning, diffusion, and ligand binding of raft lipid analogs in model and cellular plasma membranes. Biochimica et Biophysica Acta 1818, 1777–1784 10.1016/j.bbamem.2012.03.007 (2012). 22450237

[b26] Wu C. *et al.* Arp2/3 is critical for lamellipodia and response to extracellular matrix cues but is dispensable for chemotaxis. Cell 148, 973–987 10.1016/j.cell.2011.12.034 (2012). 22385962 PMC3707508

[b27] Nolen B. J. *et al.* Characterization of two classes of small molecule inhibitors of Arp2/3 complex. Nature 460, 1031–1034 (2009). 19648907 10.1038/nature08231 PMC2780427

[b28] Fritzsche M., Lewalle A., Duke T., Kruse K. & Charras G. Analysis of turnover dynamics of the submembranous actin cortex. Molecular Biology of the Cell 24, 757–767 (2013). 23345594 10.1091/mbc.E12-06-0485 PMC3596247

[b29] Goley E. D. & Welch M. D. The ARP2/3 complex: an actin nucleator comes of age. Nature Reviews Molecular Cell Biology 7, 713–726 (2006). 10.1038/nrm2026 16990851

[b30] Xu K., Babcock H. P. & Zhuang X. Dual-objective STORM reveals three-dimensional filament organization in the actin cytoskeleton. Nature Methods 9, 185–188 10.1038/nmeth.1841 (2012). 22231642 PMC3304438

[b31] Wu C. *et al.* Loss of Arp2/3 induces an NF-kappaB-dependent, nonautonomous effect on chemotactic signaling. J Cell Biology. 203, 907–916 10.1083/jcb.201306032 (2013). PMC3871425 24344184

[b32] Saffman P. G. & Delbruck M. Brownian motion in biological membranes. PNAS 72, 3111–3113 (1975). 1059096 10.1073/pnas.72.8.3111 PMC432930

[b33] Fritzsche M., Thorogate R. & Charras G. Quantitative analysis of ezrin turnover dynamics in the actin cortex. Biophys J 106, 343–353 10.1016/j.bpj.2013.11.4499 (2014). 24461009 PMC3907236

[b34] Salbreux G., Charras G. & Paluch E. Actin cortex mechanics and cellular morphogenesis. Trends in Cell Biology 22, 536–545 (2012). 22871642 10.1016/j.tcb.2012.07.001

[b35] Bovellan M. *et al.* Cellular control of cortical actin nucleation. Current Biology : CB 24, 1628–1635 10.1016/j.cub.2014.05.069 (2014). 25017211 PMC4110400

[b36] Yang W. *et al.* Arp2/3 complex regulates adipogenesis by controlling cortical actin remodelling. The Biochemical Journal 464, 179–192 10.1042/BJ20140805 (2014). 25220164

[b37] Romer L. H., Birukov K. G. & Garcia J. G. N. Focal Adhesions: Paradigm for a Signaling Nexus. Circulation Research 2006, 606–616 (2006). 16543511 10.1161/01.RES.0000207408.31270.db

[b38] Chaigne A. *et al.* A soft cortex is essential for asymmetric spindle positioning in mouse oocytes. Nature Cell Biology 15, 958–966 (2013). 10.1038/ncb2799 23851486

[b39] Yi K. *et al.* Dynamic maintenance of asymmetric meiotic spindle position through Arp2/3-complex-driven cytoplasmic streaming in mouse oocytes. Nature Cell Biology 13, 1252–1258 (2011). 10.1038/ncb2320 PMC3523671 21874009

[b40] Zhou K. *et al.* Actin-related protein2/3 complex regulates tight junctions and terminal differentiation to promote epidermal barrier formation. PNAS 110, E3820–E3829 (2013). 24043783 10.1073/pnas.1308419110 PMC3791730

[b41] Wieser S. & Schutz G. J. Tracking single molecules in the live cell plasma membrane-Do’s and Don’t’s. Methods 46, 131–140 10.1016/j.ymeth.2008.06.010 (2008). 18634880

[b42] Honigmann A. *et al.* A lipid bound actin meshwork organizes liquid phase separation in model membranes. eLife 3, e01671 10.7554/eLife.01671 (2014). 24642407 PMC3957580

[b43] Pomerantsev A. L. Confidence intervals for nonlinear regression extrapolation. Chemometrics and Intelligent Laboratory Systems 49, 41–48 (1999).

