# Supplementary material for: Cortical actin networks induce spatio-temporal confinement of phospholipids in the plasma membrane – a minimally invasive investigation by STED-FCS
Source: Sci Rep. 2015 Jun 29;5:11454. doi: 10.1038/srep11454 (PMC4484492; doi:10.1038/srep11454)
Supplement: Supplementary Information [file srep11454-s1.pdf]

Supplementary Figures and Supplementary Table

Cortical actin networks induce spatio-temporal confinement of phospholipids in the plasma membrane – a minimally invasive investigation by STED-FCS

Débora M. Andrade[1,3,§,*], Mathias P. Clausen[1,2,4,§], Jan Keller[1], Veronika Mueller[1], Congying Wu[5], James E. Bear[5,6], Stefan W. Hell[1], B. Christoffer Lagerholm[2,4,*] and Christian Eggeling[1,2,*]

[1]Department of Nanobiophotonics, Max Planck Institute for Biophysical Chemistry, Am Fassberg 11, Göttingen 37077, Germany

[2]MRC Human Immunology Unit and Wolfson Imaging Centre Oxford, Weatherall Institute of Molecular Medicine, University of Oxford, Headley Way, Oxford OX3 9DS, UK

[3]Centre for Neural Circuits and Behaviour, University of Oxford, Mansfield Road, Oxford OX1 3SR, UK

[4]MEMPHYS - Center for Biomembrane Physics, University of Southern Denmark, Campusvej 55, Odense MDK-5230, Denmark

[5]Department of Cell & Developmental Biology, and Lineberger Comprehensive Cancer Center, University of North Carolina, Chapel Hill NC 27599, USA

[6]Howard Hughes Medical Institute, University of North Carolina, Chapel Hill NC 27599, USA

[§]D.M.A. and M.P.C. contributed equally to this work

[*]Corresponding authors:

Christian Eggeling; christian.eggeling@rdm.ox.ac.uk;

B. Christoffer Lagerholm; christoffer.lagerholm@imm.ox.ac.uk;

Débora M. Andrade: debora.andrade@cncb.ox.ac.uk

Keywords: lipid diffusion, cytoskeleton, Arp2/3 complex, plasma membrane organisation, STED-FCS

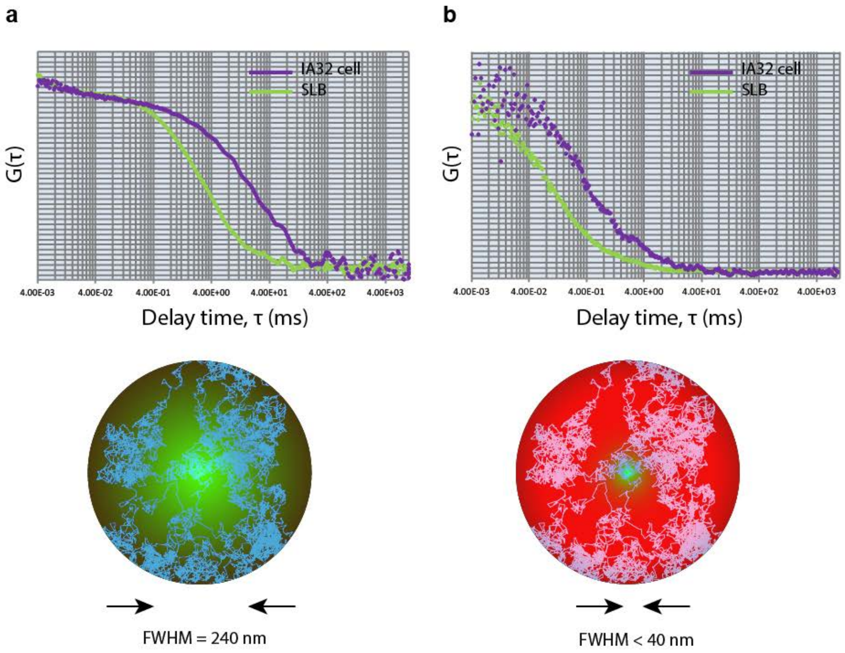

Figure S1 Assessing diffusion coefficients via FCS measurements. FCS allows the determination of the diffusion coefficient of molecules by fitting the experimental time auto-correlation curve (a, b upper panel, curves generated by the observed time-fluctuating fluorescence signal of labelled molecules diffusing through the focal spot) to a model of two-dimensional diffusion, which considers also dark states of the fluorophores used. The fitting of time auto-correlation curves determines the average time t_D that molecules take to diffuse through the focal spot. In order to obtain diffusion coefficients D from the fitted transit times t_D, we divided the focal area (whose diameter is the full-width-at-half-maximum, FWHM) by the correspondent transit time. In order to illustrate the assessment of compartmentalised diffusion by FCS, we consider experimental FCS curves from Atto647N-labelled DPPE in IA32 cells (purple) and in supported lipid bilayers (SLBs, yellow), with a FWHM of 240 nm (a) and 40 nm (b). In supported lipid bilayers, diffusion is free (unhindered). The fitted transit times in a are 27.6 ms for diffusion in IA32 cells and 2.56 ms for diffusion in SLBs. In b, the fitted transit times are 0.49 ms for IA32 cells and 0.09 ms for SLBs. Whereas in A the transit time for diffusion in IA32 cells is roughly 10 times longer than in SLBs, in B this difference is reduced to 5 times. That indicates that there is a mechanism of hindered diffusion that is characteristic in larger observation areas, but is less significant in smaller observation areas. This mechanism is the compartmentalization of the plasma membrane.

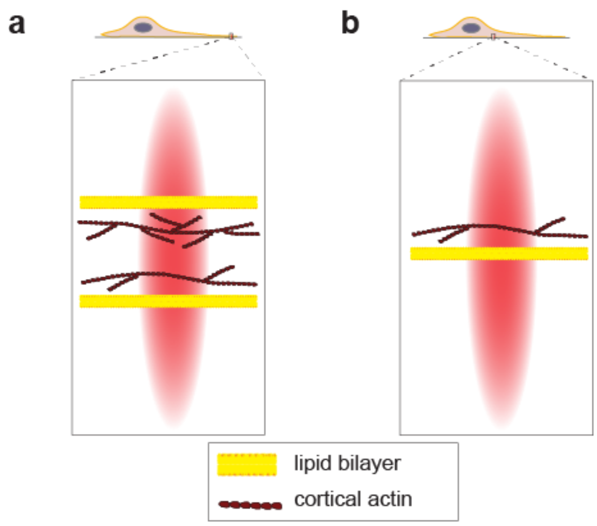

Figure S2 Schematics showing two different experimental situations of probing lipid diffusion with STED-FCS. (a) In the lamellipodia and the adjacent lamella, and close to the edge of the cells, the distance between apical and basal membranes is in the order of a few hundred nanometers. In STED-FCS experiments, the observation volume is reduced only in the x-y plane (diameter varying from 240 to 40 nm), rendering its z-dimension unaltered to approximately 700 nm. That implies that in this situation STED-FCS probed lipid diffusion in both the basal and apical membranes at the same time, averaging them. (b) Conversely, when the separation between the membranes is in the micrometer range (as it is the case more towards the cell body of most cells), we in this case only probed the basal membrane.

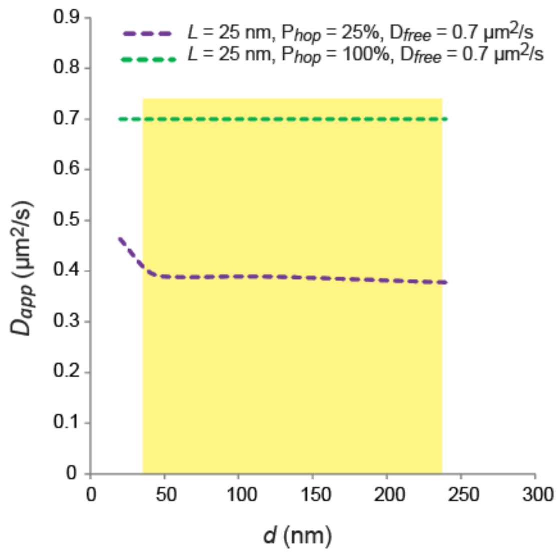

Figure S3 *In silico* STED-FCS experiments for an average compartment size L = 25 nm, free diffusion coefficient D_{free} = 0.7 μm^2/s and hopping probabilities P_{hop} = 0.25 (purple) and P_{hop} = 1 (green). The *in silico* data describes accurately the experimental FCS data for untreated (purple) and CK-666 treated (green) PtK2 cells. The curve correspondent to P_{hop} = 1 can also be obtained by setting L >> 240 nm. The region in yellow corresponds to the accessible d range for STED-FCS experiments so far. As can be observed, very small compartment sizes preclude the observation of compartmentalised diffusion.

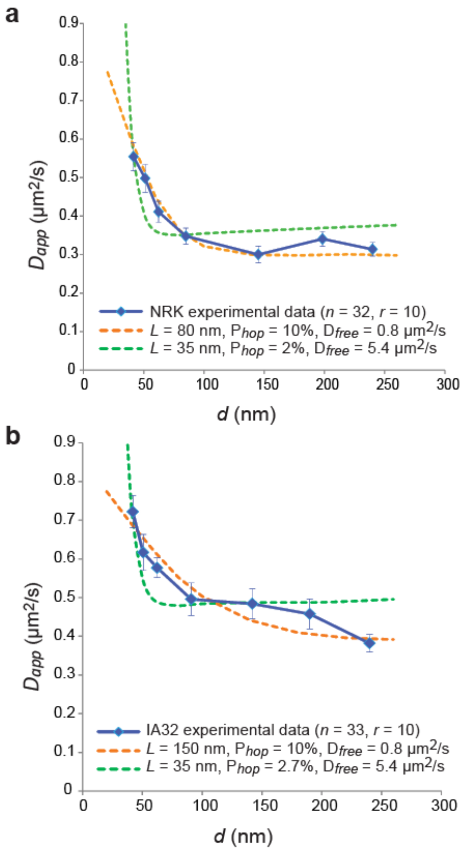

Figure S4 Comparing simulation parameters for phospholipid diffusion in cells. (a, b) Experimental DPPE diffusion coefficient for diffusion in the plasma membrane of NRK and IA32 cells, respectively, and *in silico* experiments based on diffusion through a heterogeneous lattice. In both cases, the blue solid line corresponds to the experimental data, the orange dashed line corresponds to the curve that best fitted our experimental data (without pre-defined constraints), and the green dashed line corresponds to the best fit obtained with the constraint D_{free} = 5.4 μm^2/s. In all plots, *n* stands for the number of measured cells, from *r* samples.

Table S1 Complete list of *P* values for comparisons between results from STED-FCS experiments, as determined by two-tailed unpaired *t* test. (A) NRK cells. (B) IA32 cells.

A

**Experiment**	*P* value for comparison between data representing *d* ~ 40 nm and *d* = 240 nm in the same experiment	*P* value for comparison between data representing *d* = 240 nm and control experiment (*d* = 240 nm data)
Untreated NRK cells (control - lamellipodia)	< 0.0001	N/A
Cholesterol Oxidase treated NRK cells	= 0.0105	= 0.1871
Blebbistatin treated NRK cells	< 0.0001	= 0.4113
Latrunculin B treated NRK cells	= 0.0563	< 0.0001
CK-666 treated NRK cells	= 0.8741	< 0.0001
Untreated NRK cells (cell body)	= 0.0002	= 0.0224

B

**Experiment**	*P* value for comparison between data representing *d* ~ 40 nm and *d* = 240 nm in the same experiment	*P* value for comparison between data representing *d* = 240 nm and control experiment (*d* = 240 nm data)
Untreated NRK cells (control - lamellipodia)	< 0.0001	N/A
Cholesterol Oxidase treated NRK cells	< 0.0001	= 0.6046
Blebbistatin treated NRK cells	= 0.0005	= 0.6048
Latrunculin B treated NRK cells	= 0.0582	= 0.0210
CK-666 treated NRK cells	= 0.4195	< 0.0001
Untreated NRK cells (cell body)	= 0.1486	= 0.0190
